# Modulation of microRNA processing by 5‐lipoxygenase

**DOI:** 10.1096/fj.202002108R

**Published:** 2020-11-17

**Authors:** Stella Uebbing, Marius Kreiß, Friederike Scholl, Ann‐Kathrin Häfner, Duran Sürün, Ulrike Garscha, Oliver Werz, Devaraj Basavarajappa, Bengt Samuelsson, Olof Rådmark, Beatrix Suess, Dieter Steinhilber

**Affiliations:** ^1^ Department of Biology Technical University Darmstadt Germany; ^2^ Institute of Pharmaceutical Chemistry Goethe University Frankfurt/Main Germany; ^3^ Division of Physiological Chemistry II, Department of Medical Biochemistry and Biophysics Karolinska Institute Stockholm Sweden; ^4^ Medical Systems Biology UCC, Medical Faculty Carl Gustav Carus, TU Dresden Dresden Germany; ^5^ Department of Pharmaceutical/Medicinal Chemistry, Institute of Pharmacy Friedrich Schiller University Jena Germany

**Keywords:** cancer, Dicer, inflammation, leukotriene, miRNA, 5‐lipoxygenase

## Abstract

The miRNA biogenesis is tightly regulated to avoid dysfunction and consequent disease development. Here, we describe modulation of miRNA processing as a novel noncanonical function of the 5‐lipoxygenase (5‐LO) enzyme in monocytic cells. In differentiated Mono Mac 6 (MM6) cells, we found an in situ interaction of 5‐LO with Dicer, a key enzyme in miRNA biogenesis. RNA sequencing of small noncoding RNAs revealed a functional impact, knockout of 5‐LO altered the expression profile of several miRNAs. Effects of 5‐LO could be observed at two levels. qPCR analyses thus indicated that (a) 5‐LO promotes the transcription of the evolutionarily conserved miR‐99b/let‐7e/miR‐125a cluster and (b) the 5‐LO‐Dicer interaction downregulates the processing of pre‐let‐7e, resulting in an increase in miR‐125a and miR‐99b levels by 5‐LO without concomitant changes in let‐7e levels in differentiated MM6 cells. Our observations suggest that 5‐LO regulates the miRNA profile by modulating the Dicer‐mediated processing of distinct pre‐miRNAs. 5‐LO inhibits the formation of let‐7e which is a well‐known inducer of cell differentiation, but promotes the generation of miR‐99b and miR‐125a known to induce cell proliferation and the maintenance of leukemic stem cell functions.

**Abbreviations**
Ago: Argonaute
CML: chronic myeloid leukemia
dsRBD: double‐stranded RNA‐binding domain
FLAP: 5‐LO activating protein
5‐LO: 5‐lipoxygenase
5LOΔ: 5‐LO knockout
miRNA: micro‐RNA
MM6: Mono Mac 6 cell line
PAZ domain: Piwi‐Argonaute‐Zwille domain
PLA: proximity ligation assay
RISC: RNA‐induced silencing complex
TGFβ: transforming growth factor β

## 1 INTRODUCTION

MiRNAs are a class of noncoding RNAs which modulate the expression of most of the human protein‐coding genes by binding to specific target sites in the 3′ untranslated region of their target mRNA, resulting in degradation or translational repression. Their biogenesis is tightly regulated on multiple levels and dysregulation causes many human diseases.^1^ MiRNA biosynthesis involves processing of the primary miRNA (pri‐miRNA) transcript in the nucleus by the microprocessor complex consisting of Drosha and DGCR8. The resulting ~65 nucleotides long precursor miRNA (pre‐miRNA) which forms a hairpin structure is translocated to the cytoplasm via exportin 5 and further processed by Dicer to the ~22 nucleotides long mature miRNA duplex. The functional strand of the duplex is loaded onto an argonaute (AGO) protein to form the RNA‐induced silencing complex (RISC). The miRNA guides RISC to the 3′ untranslated region of target messenger RNAs (mRNAs) by base pairing, while the AGO protein recruits factors to induce translational repression, mRNA deadenylation, and subsequent mRNA decay.^1^

Dicer is a multidomain RNase III‐type endonuclease with a size of ~220 kDa comprising 1922 amino acids. The enzyme consists of an N‐terminal helicase domain, a DUF283 (domain of unknown function), PAZ (Piwi‐Argonaute‐Zwille) domain, two RNase III (RNase IIIa/b) domains, and a C‐terminal double‐stranded RNA‐binding domain (dsRBD). The helicase domain interacts with the stem‐loop of the pre‐miRNA and the PAZ domain binds to its end, while the two RNase III domains dimerize to form the nuclease core. The region between the PAZ and the RNase III domains functions as a “molecular ruler” and facilitates production of small RNAs of a distinct size.^1^, ^2^, ^3^, ^4^ Various proteolytic cleavages of Dicer have been described, we found recently that in monocytic cells an apparently constitutive proteolysis resulted in ≈50 and ≈170 kDa Dicer fragments.^5^

Dicer enzymatic cleavage activity can be modulated by several cofactors and RNA‐binding proteins.^6^, ^7^ For instance, Dicer interacts with PACT and TRBP (TAR RNA‐binding protein) which modulate its processing efficiency and adjust the length of mature miRNAs. However, the exact role of PACT remains unclear and these cofactors seems not to be essential for Dicer‐mediated pre‐miRNA processing.^1^ The RNA‐binding protein KSRP (KH‐type splicing regulatory protein) interacts with the terminal loop of various pre‐miRNAs and promotes Dicer‐mediated cleavage.^8^ In contrast to KSRP, LIN28 blocks Dicer processing by binding to the terminal loop of some members of the let‐7 family, inducing their oligouridylation.^9^ Furthermore, Dicer‐derived miRNA products can also regulate the enzyme via a negative feedback loop. A binding site for the let‐7a miRNA is located within the human DICER1 mRNA 3’UTR, consequently overexpression of let‐7a downregulates Dicer levels.^10^

Another protein that was identified to interact with human Dicer is 5‐lipoxygenase (5‐LO), an enzyme crucial for the biosynthesis of leukotrienes which are pro‐inflammatory lipid mediators.^11^, ^12^ 5‐LO catalyzes the conversion of arachidonic acid into leukotriene A_4_, which in turn is either converted to leukotriene B_4_ or to the cysteinyl leukotrienes, leukotriene C_4_, D_4_, and E_4_. Leukotrienes are involved in the host defense, as well as in acute and chronic inflammation, and the 5‐LO pathway might also play a role in cancer development.^13^ The pro‐inflammatory enzyme is mainly expressed in immune competent cells such as monocytes, macrophages, mast cells, neutrophils, granulocytes, dendritic cells, and B‐lymphocytes, and its expression increases upon myeloid cell differentiation.^14^ However, also hematopoietic stem and progenitor cells (HSPC) were shown to express 5‐LO.^15^ First hints that 5‐LO might be involved in the regulation of myeloid cell differentiation and stem cell maintenance came from the observation that 5‐LO is required for the aberrant self‐renewal capacity of leukemic stem cells in a BCR/ABL chronic myeloid leukemia model (CML), knockout of 5‐LO prevented CML development in the mice.^16^ Subsequently, it was found that 5‐LO is strongly upregulated in AML1/ETO‐positive AML and that loss of 5‐LO expression impaired cellular dysregulation caused by oncogenic fusion proteins (RUNX1‐ETO9a, MLL‐AF9, and PML‐RARα).^17^ A key role of 5‐LO for the maintenance of leukemic stem cells was also shown in a PML/RARα‐positive stem cell model of acute myeloid leukemia.^15^

5‐LO itself and proteins associated with the 5‐LO pathway were found to be miRNA targets. For example, miR‐135a and miR‐199a‐5p downregulate the 5‐LO activating protein (FLAP), whereas miR‐219‐2 functions as a regulator of 5‐LO in macrophages.^18^, ^19^ MiR‐193b‐5p inhibited 5‐LO expression in rat cerebral ischemia and 5‐LO is a direct target of miR‐19a‐3p and miR‐125b‐5p.^20^, ^21^

It was previously demonstrated that the C‐terminal 140 amino acids of Dicer (the double‐stranded RNA‐binding domain, dsRBD) interact with 5‐LO in a cell‐free environment and in transfected human cells.^11^ These results provided a first line of evidence for a link between miRNA processing and 5‐LO. However, a detailed functional analysis of the Dicer‐5‐LO interaction and consequently a possible modulation of the miRNA biogenesis by 5‐LO remained unexplored. Here, we demonstrate the interaction of Dicer and 5‐LO in situ and its functional impact on the expression level of miRNA let‐7e. Our results suggest that 5‐LO modulates expression of distinct miRNA clusters on the pri‐miRNA level as well as the processing of let‐7e by Dicer. This implicates another noncanonical function of an enzyme involved in lipid mediator biosynthesis, that is, to regulate gene expression by modulating miRNA processing.

## 2 METHODS

### 2.1 5‐LO knockout and knockdown generation

The production of the lentivirus and the subsequent lentiviral transduction was performed as previously described.^22^ Lentiviral particles were created by polyethylenimine‐based co‐transfection of HEK293T cells with a CRISPR/Cas9 and sgRNA containing vector (pLentiCRISPRv2‐5LO), a lentiviral gag/pol packaging vector (CMVDR8.91), and an envelope plasmid encoding the glycoprotein of vesicular stomatitis virus (VSV‐G) (pMD2.G, Addgene #12259). The resulting supernatant was sterile filtered (0.45‐μm pore‐size PVDF‐membrane filter; Millipore) and concentrated (100‐fold) by ultracentrifugation over a 20% (w/v) sucrose cushion (50 000*g*, 2 hours, 4°C) for transduction that was performed for 3 days with 50 µL lentivirus on 2 × 10^6^ Mono Mac 6 (MM6) cells. The transduced cells were then selected using 2 µg/mL puromycin (Thermo Fischer Scientific) for 1 week. For the generation of single cell clones the cells were counted and diluted to a concentration of 0.3 cells per well. Cells were seeded in a 96‐well round bottom plate (Greiner Bio‐One) and screened by microscopy for wells containing more than one cell. Only wells containing single cells were expanded for several weeks until a sufficient number of cells could be harvested for storage and validation. Cell clones without any 5‐LO signal were further analyzed on genomic level using Sanger sequencing of a PCR fragment spanning the guide RNA‐binding site at ALOX5 exon 2 (Figure S1A). The absence of the 5‐LO protein was verified by performing Western blots using an anti‐5‐LO antibody (monoclonal mouse anti‐5‐LO antibody 6A12 from in‐house production) (Figure S1B). Off‐target analysis revealed that the 5‐LO sgRNA is selective for 5‐LO with at least four mismatches to other genes (Table S1). Clones 3 and 15 which exhibit a frameshift on both chromosomes (denoted 5LOΔ MM6 cells) were used for the experiments. Since the results were comparable both clones were used throughout the study. Reagents were obtained from Sigma‐Aldrich if not stated otherwise. 5‐LO knockdown MM6 cells were prepared by infection of MM6 cells with lentivirus expressing shRNA against 5‐LO (NM_000698.1‐1039s1c1) as described. Knockdown efficiency is shown in Figure S1C, and in ref.^23^ Control knockdown cells were obtained with lentivirus derived from the pLKO.1‐puro nontarget shRNA vector (Sigma‐Aldrich SHC002).

### 2.2 Cell culture

Wild‐type (DSMZ, ACC 124), 5‐LO knockdown MM6 cells,^23^ and 5‐LO knockout MM6 cells were grown in glutamine containing RPMI 1640 medium (supplemented with 10% (v/v) heat inactivated FCS, 10 µg/mL insulin, 1 × MEM nonessential amino acids, 1 mM sodium pyruvate, 1 mM oxaloacetate, 100 U/mL penicillin, and 100 µg/mL streptomycin) at 37°C and 5% CO_2_. For differentiation experiments, cells were cultured with 1 ng/mL transforming growth factor β (TGF‐β) (Peprotech) and 50 nM calcitriol for 96 hours.

### 2.3 Sequencing of small noncoding RNAs

Total RNA was extracted with TRIzol (Invitrogen) and treated with DNase I (Invitrogen) according to the manufacturer's protocol. The cDNA libraries were generated from 1 µg total RNA using the small RNA‐Seq Library Prep Kit (Lexogen) according to the manufacturer's instructions. Then, the libraries were mixed equimolarly and size selected for pre‐ and mature miRNA (130‐260 bp including 126 bp linkers) by agarose gel electrophoresis. The RNA and cDNA quality and purity were verified using the Agilent 2100 bioanalyzer (Agilent). Finally, single end sequencing was performed on a NextSeq500 (Illumina) with a read length of 75 bp. The obtained sequences were analyzed with omiRas,^24^ a web tool for annotation and differential expression analysis of noncoding RNAs derived from small RNA sequencing experiments. For proper annotation of the sequences to the human genome (hg19 UCSC) reads have been trimmed for the adapters of the small RNA‐Seq Library Prep Kit (Lexogen).

### 2.4 Analysis of primary and mature miRNAs

Total RNA isolation was carried out with the miRNeasy Mini Kit including DNase digestion (Qiagen) according to the manufacturer's protocol. The miScript II RT Kit (Qiagen) and the miScript SYBR Green Kit (Qiagen) were used for reverse transcription and qPCR analysis of mature miRNA according to the manufacturer's instructions. RNU6 served as an endogenous control. To analyze primary miRNA, total RNA was reverse transcribed using the High‐Capacity RNA‐to‐cDNA Kit (Applied Biosystems) and qPCR was performed using Fast SYBR Green PCR Master Mix (Applied Biosystems) according to the manufacturer's protocol. β‐Actin served as an endogenous control. All qPCR experiments were carried out on a StepOnePlus device (Applied Biosystems). The primer sequences are: β‐actin fwd CGGGACCTGACTGACTACCTC, β‐actin rev CTTCTCCTTAATGTCACGCACG, pri‐let‐7a‐1_fwd GTTTTGGGTGGTCTTGGAGA, pri‐let‐7a‐1_rev TGAGGGCAAAGCTGAAATCT, pri‐let‐7a‐2_#1_fwd AGGGGAAGGGCAGTAAGTGT, pri‐let‐7a‐2_#1_rev TGACCCCCAGAATAAGAACG, pri‐let‐7a‐2_#2_fwd TACCCATTCCATTTCCTCCA, pri‐let‐7a‐2_#2_rev TTGAGGCTCCCTCAGAGTGT, pri‐let‐7a‐2_#3_fwd GTGCTGGAGCAGGAGATAGG, pri‐let‐7a‐2_#3_rev CAGCCTTTACTGGGCAAAAC, pri‐let‐7a‐3_fwd GTCACAGGGCTGCGAGTATT, pri‐let‐7a‐3_rev TGTCTGCCTTCAGATTGTGC, pri‐let‐7c_fwd TGAGGCTGAATGCAATTGACT, pri‐let‐7c_rev GGCAGCCATACACCTAAGGG, pri‐let‐7e_fwd CCTCCTTCCCCTGAAATCTG, pri‐let‐7e_rev GGGGCAGAGACCTAGAAAGC, pri‐let‐7f‐2_fwd GGACAGAGTTGCAGTCAGGA, pri‐let‐7f‐2_rev CGACTGGCTCTGTTCAGGTT, gRNA‐5LO_fw CACCGTGGATCACCGGCGATGTCG, gRNA‐5LO rev AAACCGACATCGCCGGTGATCCAC, sgRNA‐5LO (with PAM) TGGATCACCGGCGATGTCGAGG.

### 2.5 Immunofluorescence microscopy and proximity ligation assay

Differentiated MM6 cells were seeded onto poly‐d‐lysine coated coverslips (Kleinfeld Labortechnik), and cultured for 30 minutes at 37°C. Cells were fixed with 4% of paraformaldehyde solution and permeabilized with acetone and 0.25% of triton X‐100 before blocking with nonimmune goat serum (Invitrogen). Samples were incubated with rabbit polyclonal anti‐dicer (Bethyl lab) (1:1000) and mouse 6A12 anti‐human‐5‐LO antibody (Goethe University Frankfurt) (1:1000). Dicer and 5‐LO were stained with Alexa Fluor 488 goat anti‐rabbit (1:1000) (Invitrogen) and Alexa Fluor 555 goat anti‐mouse antibody (Invitrogen) (1:1000). DAPI (Invitrogen) was used to stain the nucleus. Image acquisition was performed using an Axiovert 200M microscope (Carl Zeiss), a Plan Neofluar X100/1.30 Pol (DIC III) objective (Carl Zeiss), and an AxioCam MR camera (Carl Zeiss).

For proximity ligation assay differentiated MM6 cells were fixed, permeabilized, and incubated with primary antibodies as described for IF microscopy. The Duolink In Situ Red Starter Kit Mouse/Rabbit (Sigma‐Aldrich) was performed following the manufacturer's instructions. Nuclear DNA was stained with DAPI (Invitrogen). An Axiovert 200M microscope (Carl Zeiss), a Plan Neofluar X100/1.30 Pol (DIC III) objective (Carl Zeiss), and an AxioCam MR camera were used to analyze PLA interaction signals by immunofluorescence microscopy.

### 2.6 In vitro Dicer activity assay

Bacterial expression and purification of His‐tagged human Dicer fragment 1650‐1912 and in vitro Dicer assay were performed as previously described.^11^ Pre‐miRNAs were in vitro transcribed as pre‐miRNA‐HDV fusion controlled by a T7 promoter by run‐off transcription from a plasmid. To allow efficient in vitro transcription by T7 polymerase, the first nucleotide of the pre‐miRNA was changed to guanine residues. In order to obtain the correct secondary structure of the precursor miRNA, the corresponding nucleotides of the base pair were changed to uracil residues. The resulting pre‐miRNA sequences are: pre‐miR‐99: GACCCGUAGAACCGACCUUGCGGGGCCUUCGCCGCACACAAGCUCGUGUCUGUGGGUGCG pre‐let‐7e: GGAGGUAGGAGGUUGUAUAGUUGAGGAGGACACCCAAGGAGAUCACUAUACGGCCUCCUAGCUUUCC pre‐miR‐125a: GCCCUGAGACCCUUUAACCUGUGAGGACAUCCAGGGUCACAGGUGAGGUUCUUGGGAUCC). For cloning, two overlapping oligonucleotides were designed and cloned into a high copy vector carrying the HDV ribozyme using the restriction enzymes EcoRI and NcoRI straight after hybridization. The sequence of the T7 promoter AATTCTAATACGACTCACTATA was attached 5’ to the pre‐miRNAs. All sequences of the oligonucleotides and plasmids used are available upon request. For in vitro transcription, 100 µg HindIII‐linearized plasmid was incubated in 200 mM Tris‐HCl (pH 8.0), 20 mM magnesium acetate, 20 mM DTT, 2 mM spermidine with 6.25 μL T7 polymerase (homemade), and 4 mM each of ATP, CTP, GTP, and UTP at 37°C overnight. The resulting RNA was gel purified, dephosphorylated, and end labeled using γ‐[^32^P]ATP according to the manufacturer's instructions.

Prior addition of labeled pre‐miRNA substrates, 0.05 µM purified Dicer 1650‐1912 protein was preincubated at 21°C for 5 minutes without or with increasing amounts (0.05‐5 µM) of human recombinant 5‐LO^25^ in 20 µL reaction buffer containing 20 mM Tris‐HCl (pH 7.5), 5 mM magnesium chloride, 1 mM DTT, 1 mM ATP, and 150 µM BSA. After incubation at 37°C for 1 hour, the reaction was stopped by adding formamide loading dye containing 25 mM EDTA. The samples were analyzed by denaturing urea‐PAGE and phosphoimaging. Alkaline and T1 ladders were generated and used as size markers.

## 3 RESULTS

### 3.1 Sequencing of small noncoding RNAs reveals an influence of 5‐LO on miRNA expression

To investigate a functional impact of 5‐LO on the miRNA biogenesis we performed sequencing of small noncoding RNAs in differentiated wild‐type and 5‐LOΔ MM6 cells to screen for miRNAs, whose expression profile is changed by the 5‐LO knockout. The web‐based annotation tool omiRas^24^ aligned 1231 noncoding sequences to the human genome. Approximately 23% of the mapped noncoding RNAs were identified as mature miRNAs (Figure S2) and subjected to further analysis. The data set has been deposited to the GEO database, record GSE152901.

**FIGURE 1:**
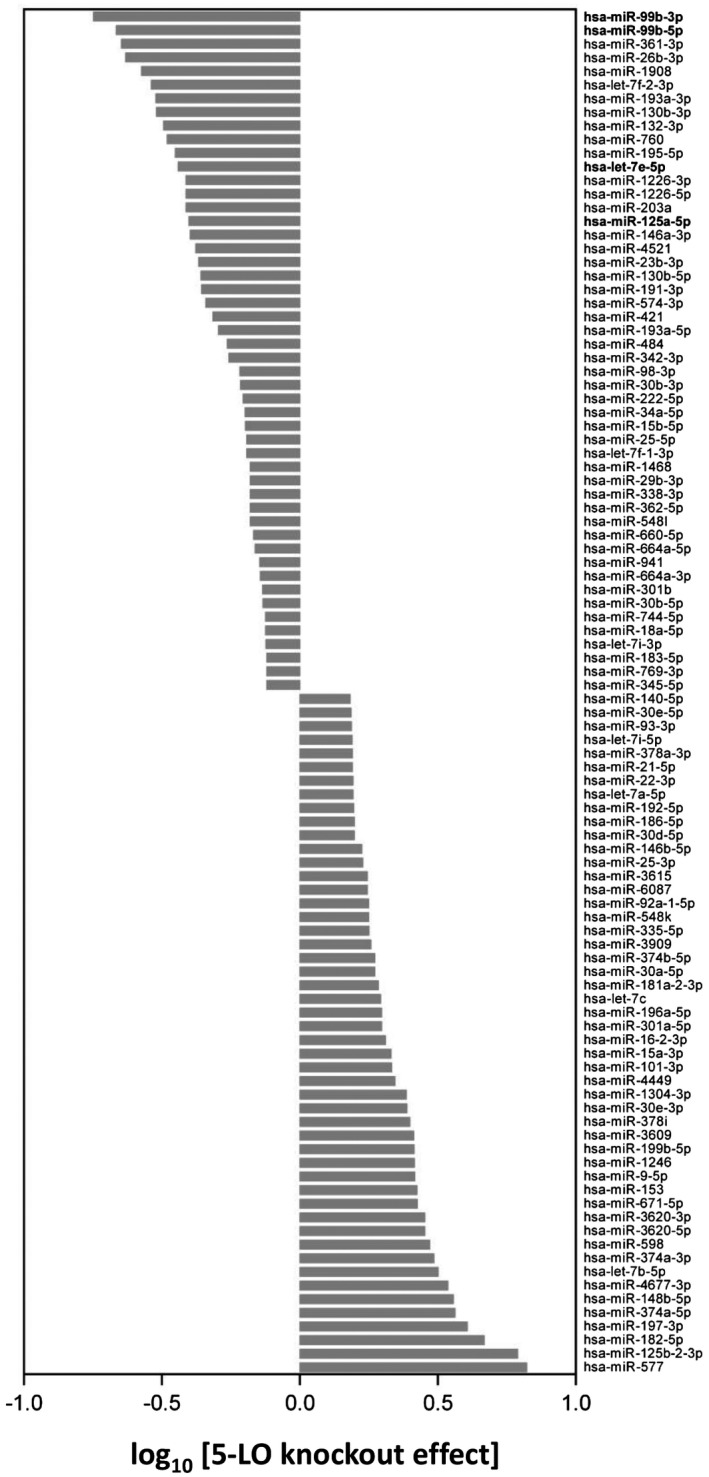
Deep sequencing of small noncoding RNAs reveals 5‐LO effects on miRNA expression. Sequencing of small noncoding RNAs was carried out on an Illumina platform in wild‐type and 5LOΔ MM6 cells which were differentiated with 1 ng/mL TGF‐β and 50 nM calcitriol for 3 days. The omiRas tool^24^ was used for analysis of the data. Differences in the miRNA expression profile between differentiated 5LOΔ MM6 cells and differentiated wild‐type cells of the 100 most influenced miRNAs are reported. The effect of 5‐LO knockout was calculated as the base 10 logarithm of the fold change of the mean of normalized counts. Results are representative for three independent experiments

Figure 1 displays the differential expression of all identified miRNAs in differentiated 5‐LOΔ MM6 cells compared to differentiated wild‐type cells. The strongest decrease by 5‐LO knockout was found for miR‐99b where similar effects were observed for both strands, that is, miR‐99b‐3p and miR‐99b‐5p (Figure 1, upper two lanes). The miR‐99b is located on chromosome 19 clustered together with the miRNAs let‐7e and miR‐125a.^26^ The expression of let‐7e‐5p and miR‐125a‐5p was also affected by 5‐LO knockout. Of note, 5‐LO knockout increased the expression of several related miRNAs, for example, let‐7a and let‐7b‐5p (Figure 1). These miRNAs, along with let‐7e‐5p, belong to the let‐7 family, which are known to influence developmental timing, tumor suppressor functions, and self‐renewal of stem cells.^27^

### 3.2 5‐LO‐dependent induction of miR‐99b‐5p and miR‐125a‐5p expression

**FIGURE 2:**
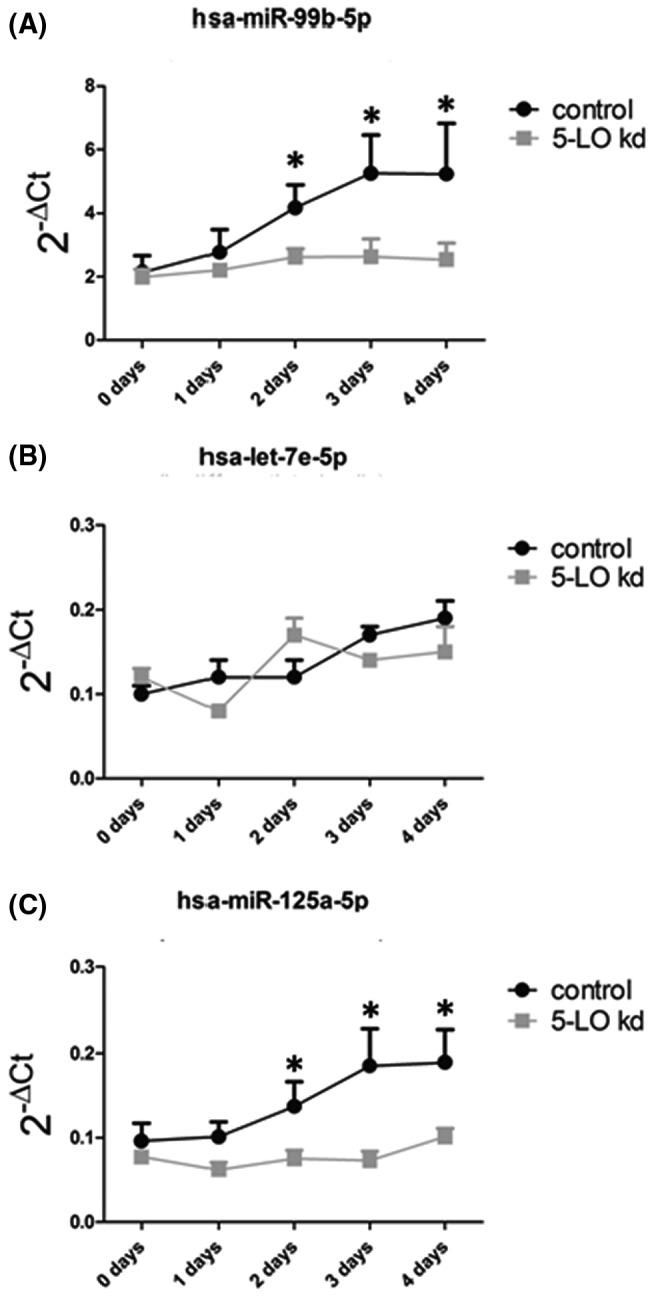
qRT‐PCR analysis of miR‐99b‐5p, let7e‐5p, and miR‐125a‐5p expression in 5‐LO knockdown and control knockdown MM6 cells. MM6 cells were differentiated with 1 ng/mL TGF‐β and 50 nM calcitriol for the indicated times, RNA was extracted, and the expression of the miRNAs was determined by qRT‐PCR. A, miR‐99b‐5p, (B) let‐7e‐5p, and (C) miR‐125a‐5p levels. The data are given as 2^−ΔCt^ values normalized to U48. Values are expressed as mean + SE, n = 4

In order to investigate 5‐LO effects on the miR‐99b/let‐7e/125a cluster, qRT‐PCR analysis was performed in undifferentiated and differentiated wild‐type and 5‐LO‐knockdown MM6 cells which we used previously for studying the 5‐LO pathway.^23^ In 5‐LO knockdown cells, expression of the three miRNAs of the cluster (miR‐99b, let‐7e, and miR‐125a) was not affected over a differentiation period of 4 days (Figure 2). In contrast, induction of MM6 cell differentiation and 5‐LO expression in wild‐type cells by calcitriol and TGF‐β significantly induced miR‐99b and miR‐125a expression (Figure 2A,C) with a time course similar to that previously described for the upregulation of 5‐LO protein in MM6 cells.^28^ Interestingly, let‐7e expression was not significantly induced during MM6 cell differentiation and showed comparable expression levels as in 5‐LO knockdown cells (Figure 2B). Thus, 5‐LO significantly increases the level of the mature miR‐99b and miR‐125a but has no effect on the third member of the cluster, let‐7e. These experiments were performed early in the study. The subsequent RNA‐Seq analysis indicated that let‐7e expression is similarly downregulated by 5‐LO knockout as miR‐99b and miR‐125a. In order to check whether this difference could be due to 5‐LO knockout (Figure 1) vs knockdown (Figure 2), the cluster was analyzed by qRT‐PCR also in the 5‐LOΔ MM6 cells.

### 3.3 5‐LO modulates the processing of distinct miRNAs

**FIGURE 3:**
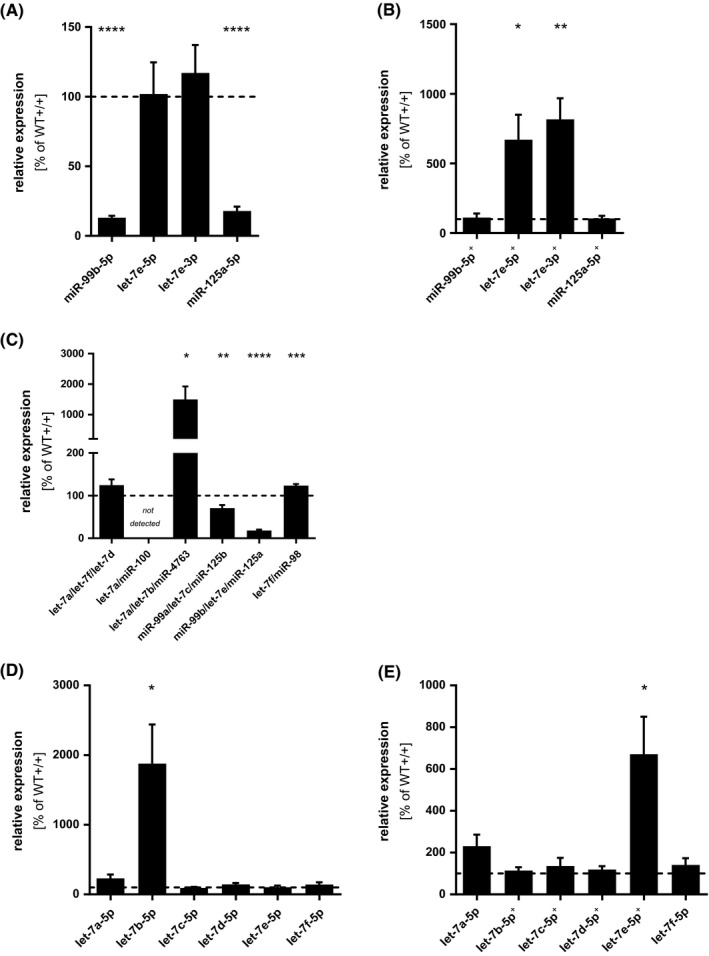
5‐LO modulates the processing of miRNAs. qRT‐PCR was used to determine the expression levels of mature miRNA (A,B,D,E) and primary miRNA (C) in wild‐type and 5LOΔ MM6 cells, differentiated with 1 ng/mL TGF‐β and 50 nM calcitriol for 4 days. The data are presented relative to differentiated wild‐type MM6 cells, set as 100%. If indicated (^+^), mature miRNA levels are also normalized to the changes of the corresponding primary miRNA levels. U6 (mature miRNA) or β‐actin (primary miRNA) was used as reference gene. Results are displayed as mean + SE and are representative for at least three independent experiments. (*P < .05, **P < .01, ***P < .001, ****P < .0001; two‐tailed unpaired *t* test)

qRT‐PCR analysis of wild‐type and 5‐LOΔ MM6 cells revealed that 5‐LO knockout strongly downregulates expression of miR‐99b‐5p and miR125a, whereas let‐7e expression is not affected (Figure 3A) confirming the results obtained with the MM6 5‐LO knockdown cells. The selective regulation of distinct miR cluster members was surprising since all three miRNAs are synthesized from the same pri‐miR‐99b/let‐7e/125a mRNA. The overall effect of 5‐LO on the miRNA expression profile includes possible 5‐LO effects at transcriptional and miRNA maturation levels. To further characterize the effects of 5‐LO, we performed qRT‐PCR experiments with differentiated wild‐type and 5LOΔ MM6 cells and compared the relative expression levels of mature miRNAs with their primary transcripts. As shown in Figure 3C, the expression of pri‐let‐7e (miR‐99b/let‐7e/miR‐125a) is reduced by the 5‐LO knockout, to 0.2‐fold compared to wild‐type cells. Thus, 5‐LO stimulates the expression of the pri‐miRNA for miR‐99b, let‐7e, and miR‐125a by about fivefold. The mature miRNA levels were normalized to the relative change of corresponding primary miRNA (Figure 3B marked with +). This normalization allows the separation of the overall effect of 5‐LO on the miRNA expression level into transcriptional effects and effects on miRNA maturation. The normalized data show that knockout of 5‐LO upregulates the maturation of miRNAs let‐7e‐5p and let‐7e‐3p (augmented 6.7‐fold and 8.2‐fold, respectively) while no effect was found for the other two cluster members miR‐99b and miR‐125a (Figure 3B). Interestingly, 5‐LO knockout did not discriminate between the let‐7e strands, the expression of let‐7e‐5p and let‐7e‐3p were similarly augmented in cells lacking 5‐LO.

In addition to the miR‐99b/let‐7e/miR‐125a cluster we analyzed also the related let‐7 miRNA family. Analysis of the mature miRNAs revealed that 5‐LO knockout strongly induces let‐7b‐5p expression (Figure 3D). This correlates with the 15‐fold upregulation of the corresponding pri‐miRNA (let‐7a/let‐7b/miR‐4763, see Figure 3C), and after normalization to the change of the pri‐miR, there was no effect of 5‐LO knockout on let‐7b‐5p (Figure 3E). No normalization could be performed for let‐7a since this miRNA is encoded by three different genes. Thus, 5‐LO knockout upregulates let‐7b expression at the pri‐miRNA level. No significant 5‐LO effects were observed on the other let‐7 miRNAs (Figure 3D,E).

5‐LO differently affects the pri‐miRNA expression of the let‐7 family members (Figure 3C). The expression levels of pri‐let‐7a‐3 (let‐7a/let‐7b/miR‐4763) and pri‐let‐7f‐2 (let‐7f/miR‐98) are elevated in cells lacking 5‐LO by 15‐fold and 1.2‐fold, respectively, suggesting that 5‐LO negatively regulates expression of these clusters. However, the expression of pri‐let‐7c (miR‐99a/let‐7c/miR‐125b) (to 0.7‐fold) and pri‐let‐7e (miR‐99b/let‐7e/miR‐125a) (to 0.2‐fold) is reduced by the 5‐LO knockout.

Taken together, 5‐LO knockout affects neither the maturation of any other let‐7 family member, nor that of miR‐99b‐5p and miR‐125a‐5p, at least under the conditions tested in this study. Thus, 5‐LO specifically inhibits the maturation of let‐7e but not the other let‐7 family members. The effects of 5‐LO on the expression of the respective pri‐miRNAs and the mature miRNAs in MM6 cells are summarized in Table 1.

**TABLE 1:** Regulation of the expression of the miR‐99b/let‐7e/125a cluster and let‐7 by 5‐LO in MM6 cells

	miR‐99b	let‐7e	miR‐125a	let‐7a	let‐7b	let‐7c	let‐7d	let‐7f
Pri‐miRNA	↑			–	↓	→	→	→
Processing	→	↓	→	→	→	→	→	→
Final outcome	↑	→	↑	↓	↓	→	→	→

Note

Changes higher than twofold (qRT‐PCR) by 5‐LO expression are indicated by up/down arrows.

It was also tested whether the modulation of the miRNA processing by 5‐LO requires an enzymatically active form of the protein. Two inhibitors of leukotriene formation (Zileuton at 1 µM, MK886 at 0.1 µM) did not interfere with the miRNA pathway modulating activity of 5‐LO. Compared to the 5‐LO knockout effects (Figure 3B) only small changes of the relative expression levels of miR‐99b‐5p, let‐7e‐5p, and miR‐125a‐5p were observed (Figure S4A,B).

Our results thus demonstrate that 5‐LO has opposing effects on the expression of the miR‐99b/let‐7e/miR‐125a cluster and on the maturation of the let‐7e miRNA (see Figure 3C,B). Finally, the expression levels of the mature miRNAs miR‐99b‐5p and miR‐125a‐5p are strongly decreased (10‐fold and 5‐fold, respectively) by the 5‐LO knockout. However, the expression level of let‐7e remains unchanged due to the counteracting impacts of 5‐LO. 5‐LO knockout decreases pri‐miR 99b/let‐7e/125a expression but increases let‐7e processing by Dicer to a similar extent. Thus, 5‐LO knockout leads to the downregulation of miRNA‐99b and ‐125a, whereas let‐7e expression is kept rather constant (compare Figure 2).

### 3.4 In situ colocalization and interaction of 5‐LO and Dicer

**FIGURE 4:**
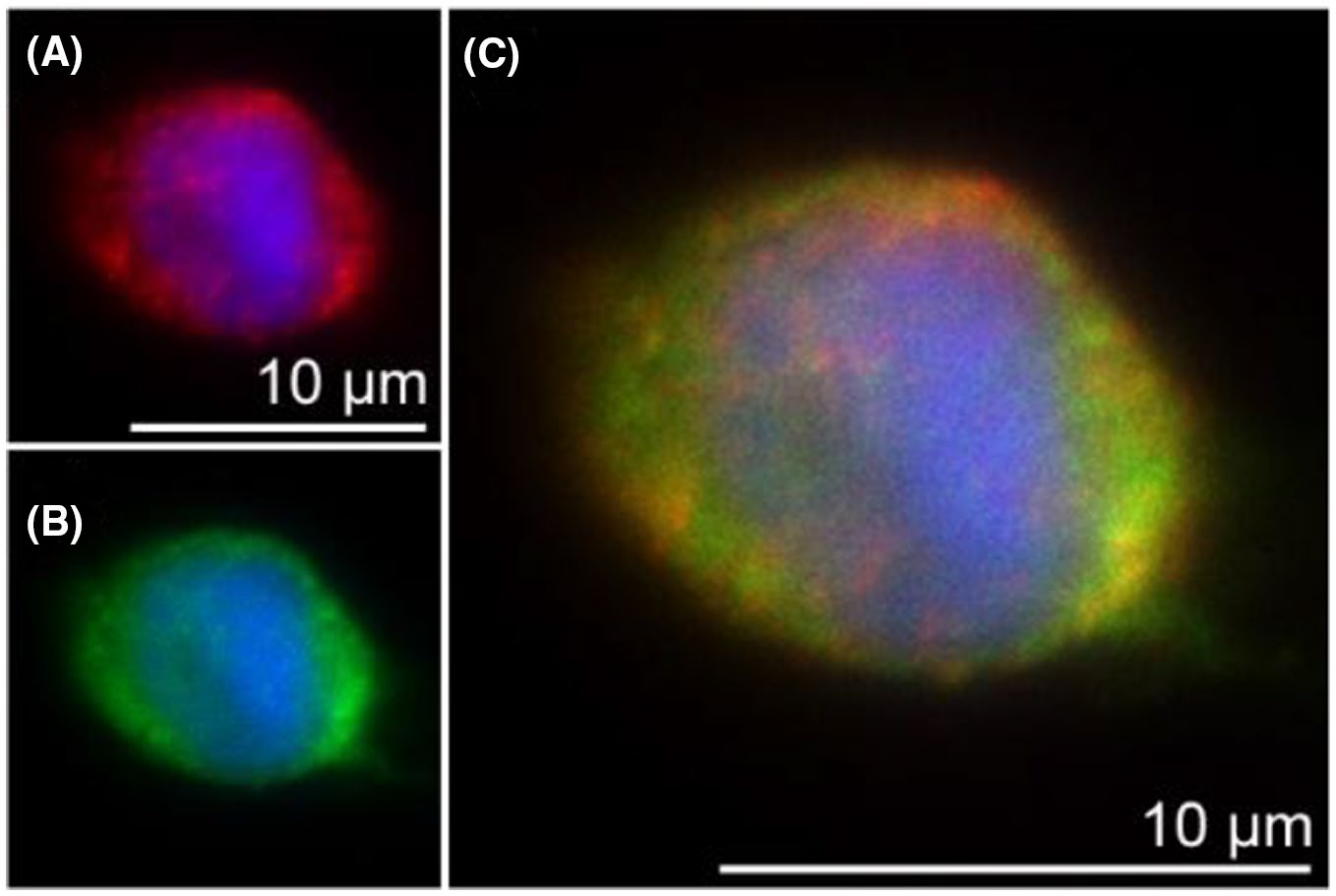
Colocalization of 5‐LO and Dicer. A‐C, Subcellular localization of 5‐LO and Dicer is monitored by indirect immunofluorescence microscopy in wild‐type MM6 cells, differentiated for 4 days with 1 ng/mL TGF‐β and 50 nM calcitriol. Cells were fixed, permeabilized, and incubated with antibodies against 5‐LO (Alexa Fluor 555, red) and Dicer (Alexa Fluor 488, green), DAPI was used to stain the nucleus. Images show single staining for 5‐LO (A), Dicer (B), or overlay of 5‐LO and Dicer (C). Results are representative for approximately 100 individual cells of three independent experiments. Scale bar represents 10 µm

The search for 5‐LO interaction partners with the yeast two hybrid system revealed Dicer as a potential candidate and subsequent studies confirmed this interaction in vitro.^11^, ^29^ In order to demonstrate the in situ interaction of 5‐LO and Dicer, we assessed their subcellular localization by immunofluorescence microscopy. 5‐LO is known to be highly expressed in differentiated myeloid cells. Therefore, we utilized the MM6 cell line which is known to exhibit a strong increase in 5‐LO expression upon 4 days treatment with the differentiation stimuli TGF‐β and calcitriol (Figure S1).^28^ Figure 4 illustrates the immunofluorescence results, showing that differentiated wild‐type MM6 cells express both 5‐LO and Dicer. In MM6 cells, both proteins are predominantly localized in the cytosol with less 5‐LO and Dicer inside the nucleus. Furthermore, an overlay of the 5‐LO and Dicer staining reveals a colocalization of both proteins within the cell (Figure 4C).

**FIGURE 5:**
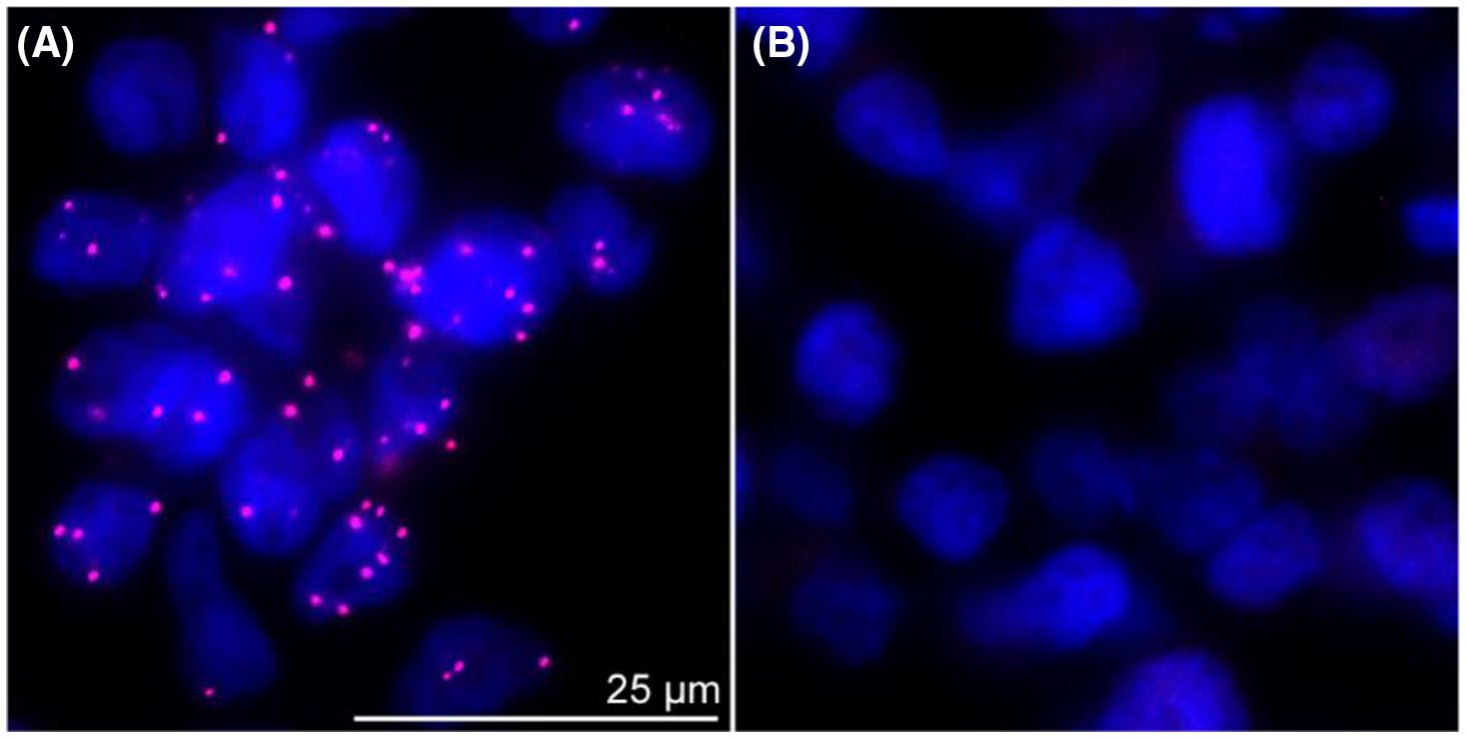
In situ interaction of 5‐LO and Dicer. A,B, In situ PLA was performed with differentiated wild‐type (A) and 5LOΔ MM6 cells (B), both differentiated with 1 ng/mL TGF‐β and 50 nM calcitriol for 4 days. Proximity probes against mouse anti‐5‐LO and rabbit anti‐Dicer were used. In situ PLA signals (magenta dots) visualize 5‐LO‐Dicer interactions and DAPI (blue) was used to stain the nucleus. Results are representative for approximately 100 individual cells of three independent experiments. Scale bar represents 25 µm

To further confirm interaction of 5‐LO and Dicer in situ we performed a proximity ligation assays (PLA), a tool that monitors protein‐protein interactions within cells if the target proteins are in close proximity (<40 nm). The method is based on oligonucleotides attached to the respective secondary antibody. If both targets are in close proximity, the oligonucleotides ligate to new circular DNA. After amplifying the circular DNA and hybridizing it with a fluorophore, positive PLA signals are detectable by fluorescence microscopy.^30^, ^31^ Wild‐type MM6 cells display strong PLA signals upon differentiation (Figure 5A), whereas no signals were detected in 5LOΔ MM6 cells (Figure 5B). These findings substantiate the 5‐LO‐Dicer interaction and exclude false‐positive PLA signals evoked by unspecific antibody binding. Taken together, our results demonstrate that an in situ interaction of 5‐LO and Dicer occurs in intact differentiated MM6 cells.

### 3.5 LPS does not modulate the 5‐LO effects on the miR‐99b/let‐7e/125a cluster

Previously, we found that in monocytic cells Dicer is constitutively cleaved into two fragments by a serine protease and that stimulation of monocytes and MM6 cells with LPS leads to upregulation of full‐length Dicer.^5^ In order to investigate whether the 5‐LO/Dicer interaction and the resulting modulation of miRNA expression depends on Dicer cleavage, wild‐type and 5LOΔ MM6 cells were differentiated and LPS (1 µg/mL) was added 6 hours before cell harvest. Then, RNA was extracted and the small noncoding RNAs were analyzed by next generation sequencing. Figure S3 shows a 2D plot where the Y‐axis illustrates the effects of 5‐LO knockout in LPS‐treated cells and the *x*‐axis displays the effects of 5‐LO knockout in cells without LPS treatment. As can be seen from Figure S3, most of the miRNAs, including miR‐99b, let‐7a, and miR‐125a locate close to the diagonal axis suggesting that LPS does not modulate the 5‐LO effects on the expression of this miRNA cluster.

### 3.6 5‐LO modulates precursor miRNA processing by Dicer 1650‐1912 in vitro

**FIGURE 6:**
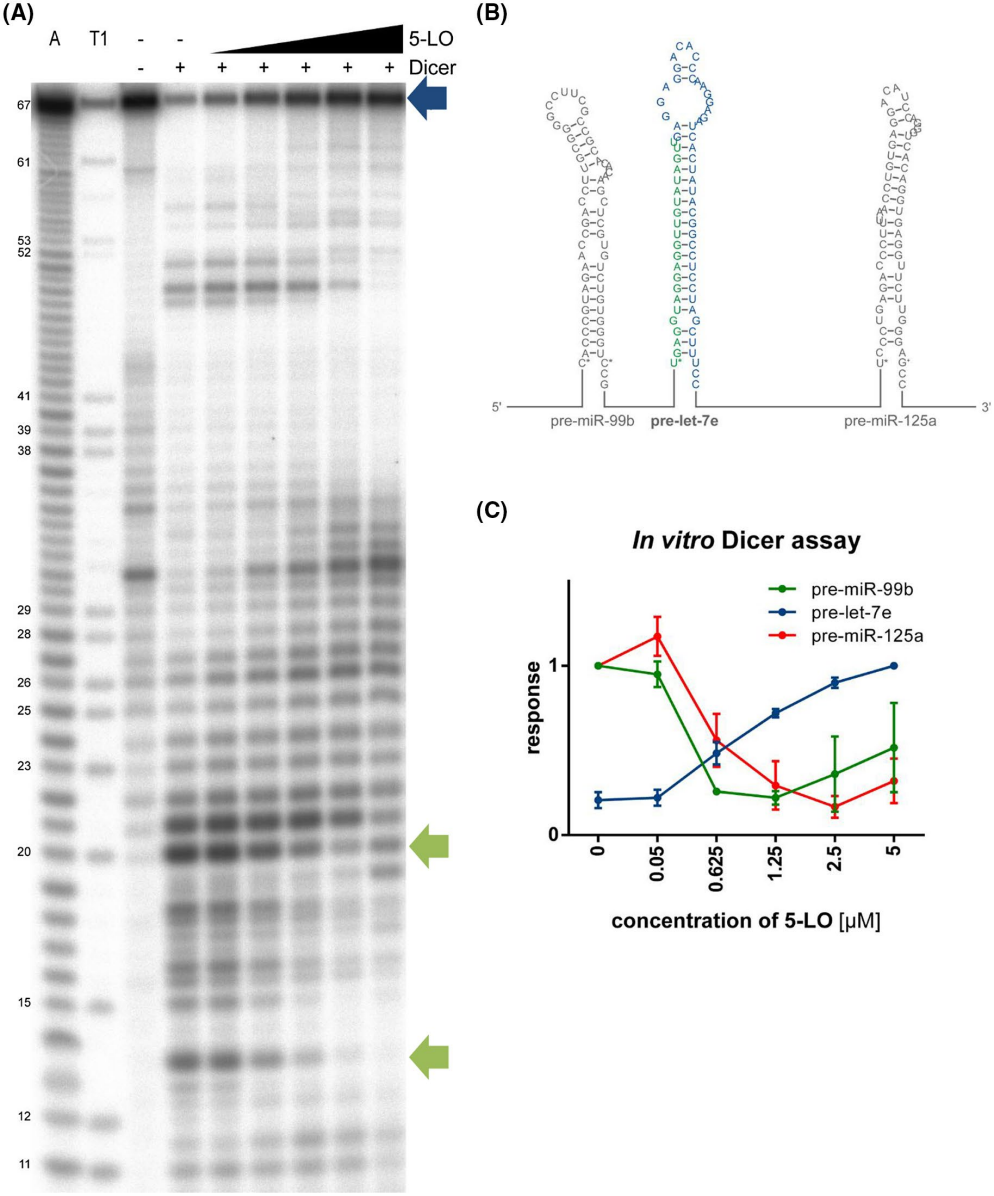
5‐LO modulates pre‐miRNA processing activity of human Dicer concentration dependently. A, Purified recombinant human Dicer 1650‐1912 fragment (0.05 µM) was incubated at 21°C for 5 minutes without or with increasing amounts of recombinant human 5‐LO (0.05, 0.625, 1.25, 2.5, and 5 µM) prior addition of ^32^P‐labeled pre‐let‐7e. After incubation at 37°C for 1 hour, samples were analyzed by denaturing PAGE and autoradiography. An alkaline (A, lane 1) and T1 ladder (T1, lane 2) were used as size markers. Full‐length pre‐let‐7e RNA is marked by a blue arrow, nucleotide positions with a decrease in band intensity are marked by green arrows. B, Schematic representation of the secondary structure of the pre‐miR‐99b/let‐7e/miR‐125a cluster. The pre‐miRNAs are shown with their exact base sequence. The sequences between the pre‐miRNAs are drawn as lines, the corresponding length is marked. C, Concentration‐response curve of the effects of 5‐LO protein on pre‐miR‐99b, pre‐let‐7e, and pre‐miR‐125a processing by Dicer 1650‐1912. For each pre‐miR substrate (compare Figures 6A and S5A,B), the values corresponding to either the lowest/highest 5‐LO concentration of the full‐length pre‐miRNA was set to 1. Results are shown as mean ± SE of three independent experiments

5‐LO seems to affect Dicer processing in a pre‐miRNA‐specific manner. To elucidate the effect of 5‐LO on pre‐miRNA processing by Dicer in a molecularly defined setting, in vitro Dicer cleavage assays were performed.^11^ Purified recombinant human Dicer 1650‐1912 fragment comprising the RNase IIIb motif and the dsRBD, which has been shown to interact with 5‐LO, was used for the enzymatic assay. This Dicer fragment is known to exhibit RNase III activity, but is not capable to produce small RNAs of one distinct size, instead several RNA fragments are formed.^11^ The organization of the pri‐miR 99b/let‐7e/125a cluster is shown in Figure 6B with the pre‐miRNA let‐7e indicated in color. Our results reveal that 5‐LO inhibits the cleavage of pre‐let‐7e by Dicer 1650‐1912 (Figure 6A lane 4‐9 and Figure 6C). The band intensity of the unprocessed precursor (blue arrow) increased with increasing 5‐LO concentrations, while products at 20 and 13 nt decreased (green arrows). We also examined the processing of the two other miRNAs of the cluster, pre‐miR‐125a and pre‐miR‐99b. In contrast to pre‐let‐7e, 5‐LO strongly enhances cleavage of pre‐miR‐125a (Figure S5, blue arrows) and slightly enhances the processing of pre‐miR‐99b concentration dependently. These two pre‐miR substrates were digested mainly to short fragments, as found before for pre‐let‐7a‐3.^11^ Thus, 5‐LO modulates in vitro Dicer RNase III activity in a pre‐miRNA‐specific manner and exerts different effects on the processing of the clustered precursors pre‐miR‐99b, pre‐let‐7e, and pre‐miR‐125a. 5‐LO inhibits pre‐let‐7e processing and stimulates processing of the pre‐miRNAs 125a and 99b.

## 4 DISCUSSION

The miRNA pathway is crucial for posttranscriptional gene silencing and the fine tuning of gene expression. MiRNAs control physiological processes such as the maintenance of tissue homeostasis, cell proliferation and differentiation, as well as immune functions, just to mention a few. To avoid dysregulation of miRNA functions which can lead to severe human diseases, their biogenesis is tightly regulated at multiple levels.^1^ Here, we describe a novel regulation of miRNA processing by the enzyme 5‐LO which is involved in the biosynthesis of leukotrienes as part of the innate immune system.^32^ However, 5‐LO has also been associated with noncanonical functions, such as regulation of p53 as well as β‐catenin activity, suggesting that 5‐LO, which in many cells is found inside the nucleus, can also act as a regulator of gene transcription.^15^, ^33^, ^34^, ^35^ 5‐LO strongly induced the expression of the pri‐miRNA of the miR‐99b/let‐7e/miR‐125a cluster. Surprisingly, in contrast to our RNA‐Seq data, we did not observe a 5‐LO‐induced increase in mature let‐7e by qRT‐PCR in 5‐LO knockdown as well as 5‐LO knockout cells. The discrepancy between the RNA‐Seq and the qRT‐PCR experiments is unclear at the moment, one possible reason could be that the RNA‐Seq analysis for mature let‐7e is unspecific. Limitations of miRNA‐Seq and the value of confirmation by other methods have been discussed.^36^

**FIGURE 7:**
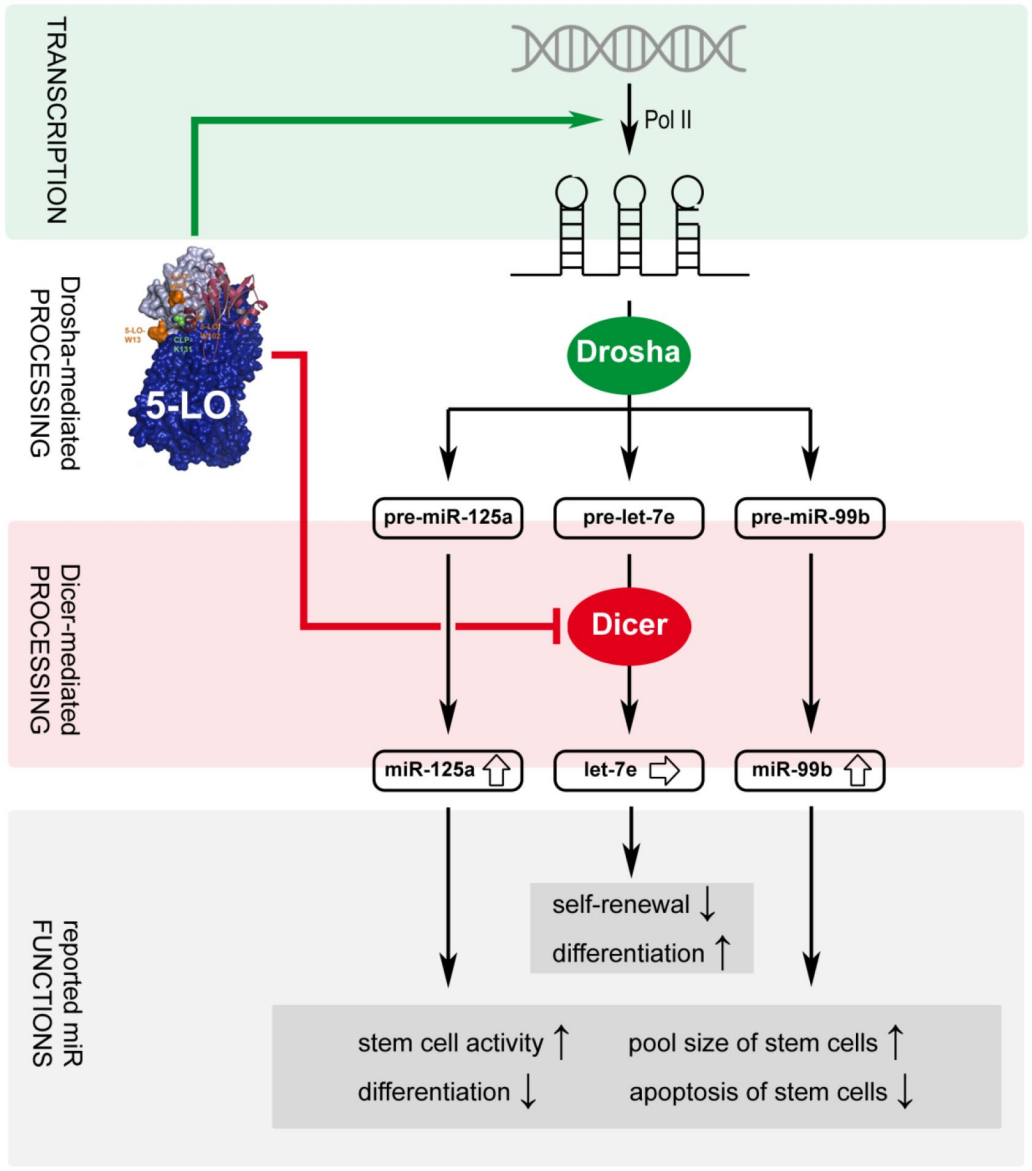
Model of the regulation of the miR‐99b/let‐7e/125a cluster by 5‐LO. 5‐LO strongly increases expression of the mRNA encoding the miR‐99b/let‐7e/125a cluster and specifically inhibits processing of pre‐let‐7e by Dicer. This leads to enhanced expression of miR‐99b and miR‐125a, whereas the let‐7e expression remains unchanged, so that 5‐LO changes the balance between let‐7e and miR‐99b/miR‐125a

The strong induction of the pri‐miRNA of the miR‐99b/let‐7e/miR‐125a cluster by 5‐LO lead to increased miR‐99b and miR‐125a levels but let‐7e expression was not affected. We observed that 5‐LO specifically inhibits the processing of pre‐let‐7e by Dicer, whereas the processing of the pre‐miRNAs‐99b and ‐125a was not inhibited but rather stimulated. Differentiation of MM6 cells with TGF‐β and calcitriol leads to the strong induction of 5‐LO expression^28^ which in turn promotes upregulation of miR‐99b and miR‐125a. However, the opposing effects of 5‐LO on the pri‐miRNA expression of the miR‐99b/let‐7e/miR‐125a cluster, and on the processing of pre‐let‐7e, result in quite constant let‐7e levels during differentiation of MM6 cells (Figure 7). This is of special interest since the miR‐99b/let‐7e/miR‐125a cluster was reported to be a pervasive regulator of the TLR pathway by directly targeting some of the mRNAs coding for proteins involved in signaling such as TLR4, CD14, and IRAK1.^37^ In cellular assays, it was found that the cluster inhibits the LPS‐dependent production of inflammatory cytokines such as TNFα and IL‐6.^37^ The cluster is known to have binding sites for SMAD, SP1, STAT3, and NFκB in the promoter which mediate responses to TGF‐β and TLR stimulation.^37^ Previous studies have shown that this miRNA cluster is involved in the coordination of the immune response and in the limitation of host defense reactions by maintaining the immune suppressive phenotype of antigen presenting cells.^38^

Moreover, a high expression of the miR‐99b/let‐7e/miR‐125a cluster in hematopoietic stem cells and its downregulation upon differentiation was observed previously.^39^ MiR‐125a alone was shown to enhance stem cell activity and to retain cells in a primitive state.^39^ Furthermore, this miRNA was also shown to amplify the hematopoietic stem cell pool size via differentiation stage‐specific reduction of the hematopoietic stem progenitor cell apoptosis.^40^ Similarly, miR‐99 was identified to regulate normal and malignant hematopoietic stem cell renewal. It promotes long‐term reconstitution activity and inhibits cell differentiation.^41^ In contrast to miR‐125a and miR‐99b, let‐7 expression was found to increase upon cell differentiation.^42^ In the same study, let‐7 was found to downregulate self‐renewal and to induce multipotent differentiation of breast cancer stem cells.^42^ Furthermore, let‐7e is upregulated during myeloblast to monocyte differentiation.^43^ Our results now demonstrate a specific regulation of the expression of the individual members of the miR‐99b/let‐7e/miR‐125a cluster by 5‐LO, leading to enhanced levels of miR‐125a and miR‐99b and unaffected levels of let‐7e in MM6 cells. Given the opposing effects on stem cell functions of miR‐125a and miR‐99b, on the one hand, and let‐7e, on the other hand, their divergent regulation by 5‐LO seems to be a mechanism to change the physiological profile of this miRNA cluster and to promote maintenance of stem cells and to suppress cell differentiation.

This is supported by the observation that 5‐LO also prominently inhibits let‐7b expression. This miRNA is a well‐known suppressor of cell proliferation in a large variety of cell types.^27^

The upregulation of miRNAs which promote stem cell proliferation and inhibit cell differentiation as well as the inhibition of several let‐7 miRNAs which promote cell differentiation fit well to the observation that 5‐LO is expressed in hematopoietic stem cell compartments and that selective inhibition of 5‐LO impairs the stem cell capacity of PML/RARα‐positive HSPCs.^15^ Furthermore, 5‐LO is required for the aberrant self‐renewal capacity of leukemic stem cells. Knockout of 5‐LO in BCR/ABL transduced bone marrow cells completely prevented the development of chronic myeloid leukemia in mice.^16^ Interestingly, a direct interaction between enzymatically inactive 5‐LO (after treatment with inhibitors) and β‐catenin caused an inhibition of the WNT pathway. This was proposed as a mechanism for the effect of the inhibitors to reduce accumulation of β‐catenin in the nucleus, and thus, maintenance of cancer stem cell‐like cells.^15^, ^44^

Previous studies investigated how the miRNA processing activity of the key enzymes Drosha and Dicer is regulated by diverse proteins. For instance, TGF‐β and BMP (bone morphogenetic protein) signaling promotes expression of miR‐21 via recruiting the Smad signal transducer to the Drosha microprocessor complex facilitating pri‐miR‐21 cleavage.^45^ Furthermore, LIN28 prevents processing of pre‐let‐7 by Dicer. The RNA‐binding protein interacts with the terminal loop of pre‐let‐7 to induce its oligouridylation.^9^ KSRP also binds to the terminal loop of several pre‐miRNAs. However, instead of blocking the cleavage by Dicer, KSRP promotes the Dicer‐mediated cleavage of those pre‐miRNAs.^8^ Our data add another regulatory mechanism by identifying 5‐LO as a regulator of the processing of distinct miRNAs by Dicer. We demonstrate that 5‐LO interacts with Dicer in MM6 cells and that 5‐LO modulates in vitro Dicer activity in a pre‐miRNA‐specific manner. On the one hand, the Dicer cleavage of pre‐let‐7e was inhibited by 5‐LO concentration dependently. On the other hand, especially the processing of pre‐miR‐125a as well as of pre‐miR‐99b was augmented by 5‐LO also in a concentration‐dependent manner. Consequently, our study suggests that 5‐LO is a miRNA‐specific regulator of Dicer activity. Previous data have shown that 5‐LO directly binds to the C‐terminal dsRNA‐binding domain of Dicer^11^ and that the dsRNA‐binding domain is located close to the RNase IIIa/b domain which cleaves the miRNA substrate.^4^ Cryo‐EM analysis of a Dicer‐TRBP‐let‐7 complex revealed that the dsRNA‐binding domain interacts with the miRNA duplex of pre‐let‐7a‐1 and it was suggested that the Dicer dsRNA‐binding domain might be involved in substrate recruitment and processing.^4^ Therefore, it is tempting to speculate that the interaction of 5‐LO with the dsRNA‐binding domain of Dicer alters the substrate recognition so that the processing of pre‐let‐7e is inhibited.

We found recently that for monocytic cells including MM6, Dicer is subjected to an apparently constitutive proteolysis resulting ≈50 and ≈170 kDa fragments. Stimulation of differentiated MM6 cells with LPS or zymosan inhibited this proteolysis leading to abundant full‐length Dicer.^5^ It has been published that recombinant Dicer fragments containing the C‐terminal part have RNase III activity, mostly leading to products shorter than ≈22 nt.^3^, ^11^, ^46^ However, limited proteolysis of intact Dicer (with proteinase K) enhanced formation of 22 nt product in vitro.^11^, ^47^, ^48^ Furthermore, when recombinant Dicer fragments were combined in vitro, this resulted in 22 nt products and it was concluded that the C‐terminal part with both RNase III domains together with a part containing PAZ and DUF283 domains is required for formation of 22 nt products.^49^ In this study, the Dicer activity producing miRNAs in MM6 cells differentiated with only TGF‐β and calcitriol could be full‐length Dicer at very low levels, or fragments combining to an active Dicer enzyme. Interestingly, LPS treatment of MM6 cells which leads to the upregulation of full‐length Dicer did not change the effects of 5‐LO knockout on the expression of the members of the miR‐99b/let‐7e/miR‐125a cluster (Figure S3) which suggests that proteolytic cleavage of Dicer does not affect 5‐LO/Dicer interaction. Accordingly, differentiation of MM6 cells in presence of LPS upregulated miR‐99b and 125a, but less so for let‐7e (compare Figures 6A and S9 in ref.^5^), indicating that the inhibitory effect of 5‐LO on processing of pre‐let‐7e occurs also in cells expressing full‐length Dicer.

A previous study demonstrated that 5‐LO is targeted directly by the miRNAs miR‐19a‐3p and miR‐125b‐5p.^20^ Considering that miR‐125b‐5p and miR‐125a‐5p share the same seed sequence at the 5’ end of the miRNAs, it is possible that also miR‐125a‐5p targets 5‐LO enabling a regulation via a negative feedback loop.^50^ Our present study suggests that 5‐LO enhances the overall expression of miR‐125a‐5p. This effect may be limited under certain physiological conditions by such a feedback loop.

In summary, our study describes a novel role of 5‐LO in regulation of miRNA biogenesis on the transcriptional and posttranscriptional levels. In particular, our findings provide a new mechanism for a precise regulation of clustered miRNAs by the divergent inference of 5‐LO with different steps of miRNA generation. Additionally, the findings suggest an unexpected link between inflammation, miRNA biogenesis, and self‐renewal of blood cells.

## COMPETING INTEREST

All authors declare no competing interests.

## AUTHOR CONTRIBUTIONS

D. Steinhilber, O. Rådmark, B. Suess, and B. Samulesson planned the study. S. Uebbing, M. Kreiss, U. Garscha, O. Werz, D. Sürün, D. Basavarajappa, D. Steinhilber, O. Rådmark, B. Suess, and B. Samulesson planned experiments and interpreted data. S. Uebbing, F. Scholl, AK. Häfner, U. Garscha, and M. Kreiss performed experiments. S. Uebbing, O. Rådmark, B. Suess, and D. Steinhilber wrote the manuscript. All authors contributed to preparing the manuscript.

## Supporting information

Supplementary Material
