# Supplementary material for: Modulation of microRNA processing by 5‐lipoxygenase
Source: FASEB J. 2020 Nov 17;35(2):e21193. doi: 10.1096/fj.202002108R (PMC12315505; doi:10.1096/fj.202002108R)
Supplement: Supplementary file 1 — Supplementary Material [file FSB2-35-e21193-s001.pdf]

Supplement

Modulation of microRNA processing by 5-lipoxygenase

Stella Uebbing[a,b,c], Marius Kreiß[b], Friederike Scholl[a,b,c], Ann-Kathrin Häfner[b], Duran Sürün[e], Ulrike Garscha[d], Oliver Werz[d], Devaraj Basavarajappa[c], Bengt Samuelsson[c], Olof Rådmark[c*], Beatrix Suess[a*], Dieter Steinhilber[b*]

[a] Department of Biology, Technical University Darmstadt, Germany

[b] Institute of Pharmaceutical Chemistry, Goethe University, Frankfurt/Main, Germany

[c] Division of Physiological Chemistry II, Department of Medical Biochemistry and Biophysics, Karolinska Institute, Stockholm, Sweden

[d] Department of Pharmaceutical/Medicinal Chemistry, Institute of Pharmacy, Friedrich Schiller University, Jena, Germany

[e] Medical Systems Biology, UCC, Medical Faculty Carl Gustav Carus, TU Dresden, Fetscherstr. 74, 01307 Dresden, Germany

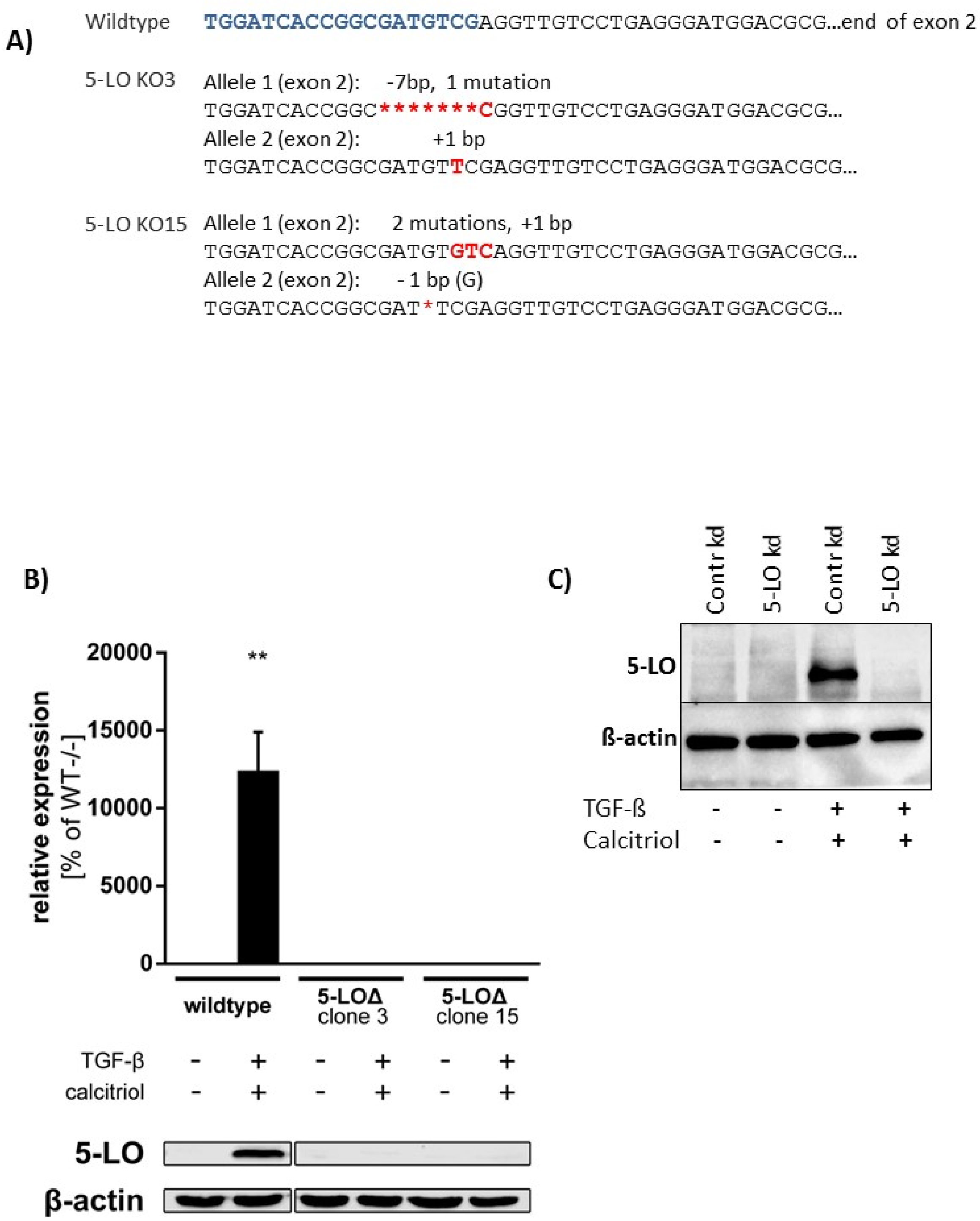

Fig. S1: 5-LO protein expression in MM6 cells. A) Sanger sequencing of the 5-LO knockout clones KO3 and KO15. Deletions are marked by * and mutations are shown in red, the sgRNA target sequence is shown in blue. B) and C) Determination of 5-LO protein expression in MM6 cells by Western blot. B) Wild type and 5-LOΔ MM6 cells (clone 3 and 15) were differentiated with 1 ng/mL TGF-β and 50 nM calcitriol for four days. β-actin was used as reference protein. The data are presented relative to undifferentiated wild type (WT-/-) MM6 cells and displayed as mean +SD. Results are representative for three independent experiments. (*$p < 0.05$, **$p < 0.01$; two-tailed unpaired t test). C) 5-LO-Knockdown: Representative Western blot of undifferentiated and differentiated MM6 cells treated with control shRNA or shRNA targeting 5-LO.

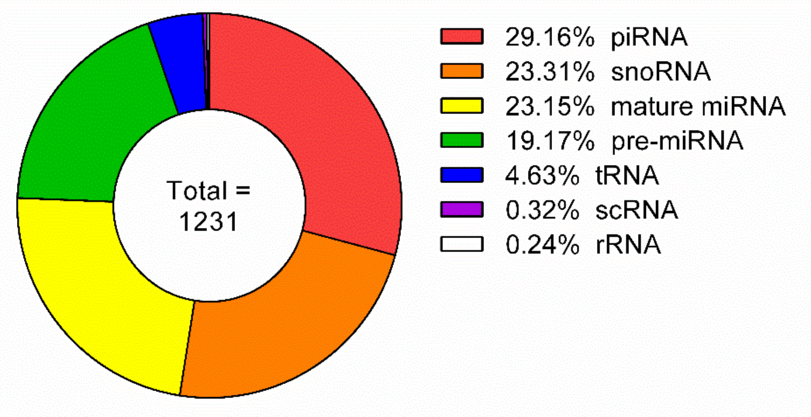

Fig. S2: Proportional distribution of the non-coding RNAs detected in the sequencing of small non-coding RNAs. The omiRas tool (24) was used to annotate the detected sequences of which 1231 could be mapped to the human genome. Proportional distribution of the different classes of non-coding RNAs is displayed.

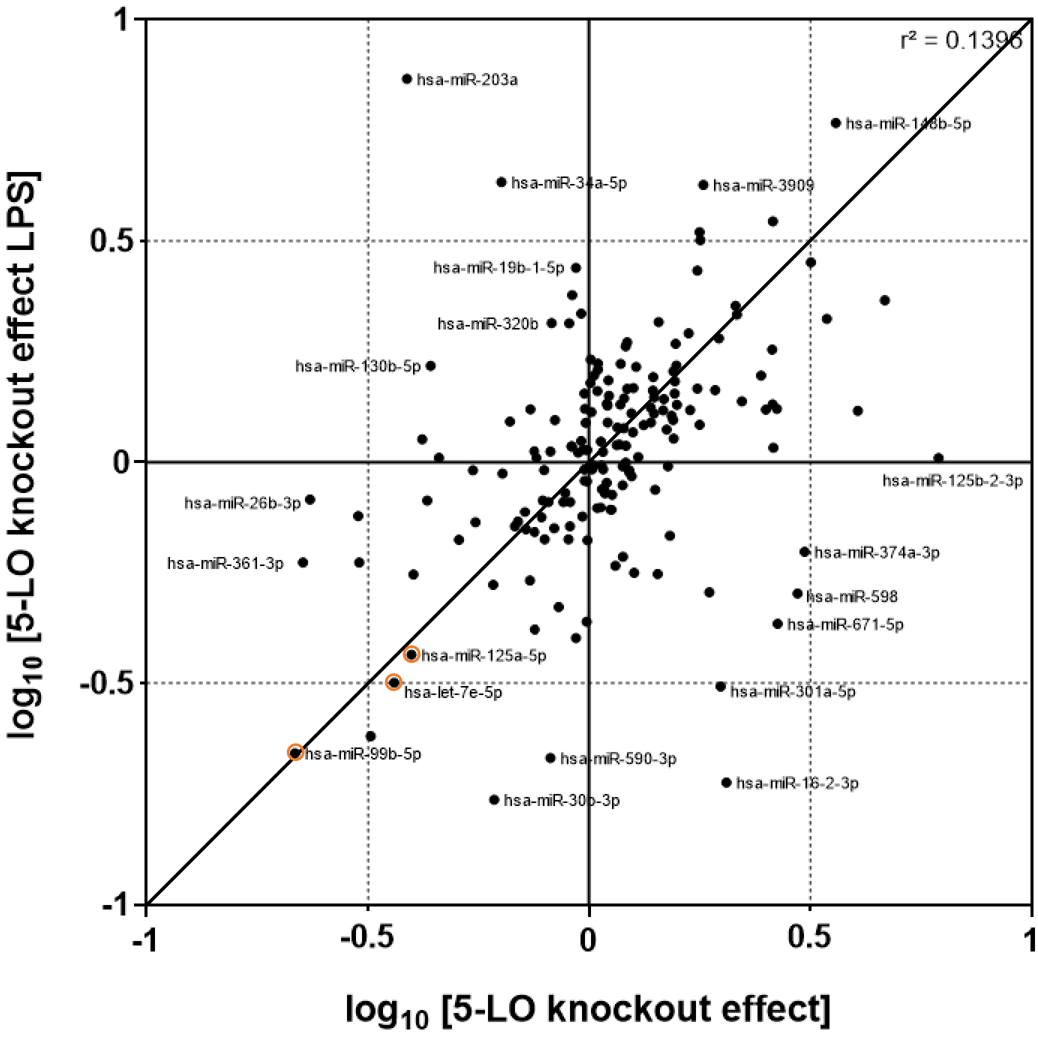

Fig. S3: Effects of 5-LO knockout on miRNA expression in MM6 cells. Sequencing of small noncoding RNAs was performed on an Illumina platform in wild type and 5-LOΔ MM6 cells, differentiated for three days with 1 ng/mL TGF-β and 50 nM calcitriol. The effect of 5-LO knockout is displayed in differentiated MM6 cells (x-axis) and in differentiated MM6 cells stimulated with 1 µg/mL LPS for the last 6 h (y-axis). The data were analyzed with the omiRas tool101. Differences in the miRNA expression profile are shown as the base 10 logarithm of the x-fold change of the mean expression of three independent experiments.

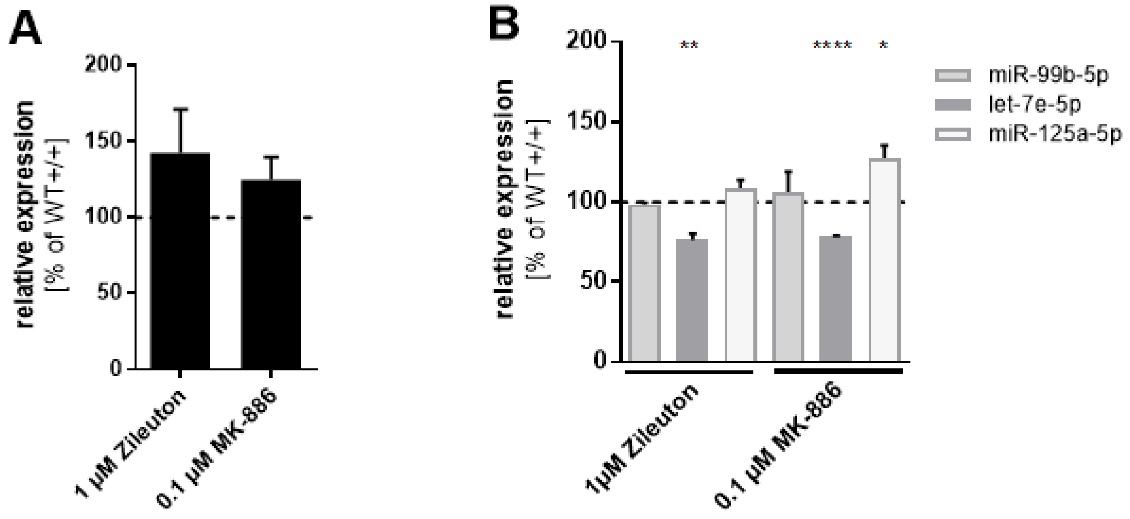

Fig. S4: 5-LO modulates the processing of miRNAs independent of its enzymatic activity. (A-B) qRT-PCR was used to determine the expression levels of pri-miRNA 99b/let-7e/125a (A) and the mature miRNA (B) in wild type MM6 (WT +/+) cells, differentiated with 1 ng/mL TGF-β and 50 nM calcitriol for four days and also treated twice (day 0 and after 48 h) with 1 µM Zileuton or 0.1 µM MK886. The data are presented relative to differentiated wild type MM6 cells, set as 100%. U6 (mature miRNA) or β-actin (primary miRNA) was used as reference gene. Results are displayed as mean + SE and are representative for at least three independent experiments. (*$p < 0.05$, **$p < 0.01$; two-tailed unpaired t test)

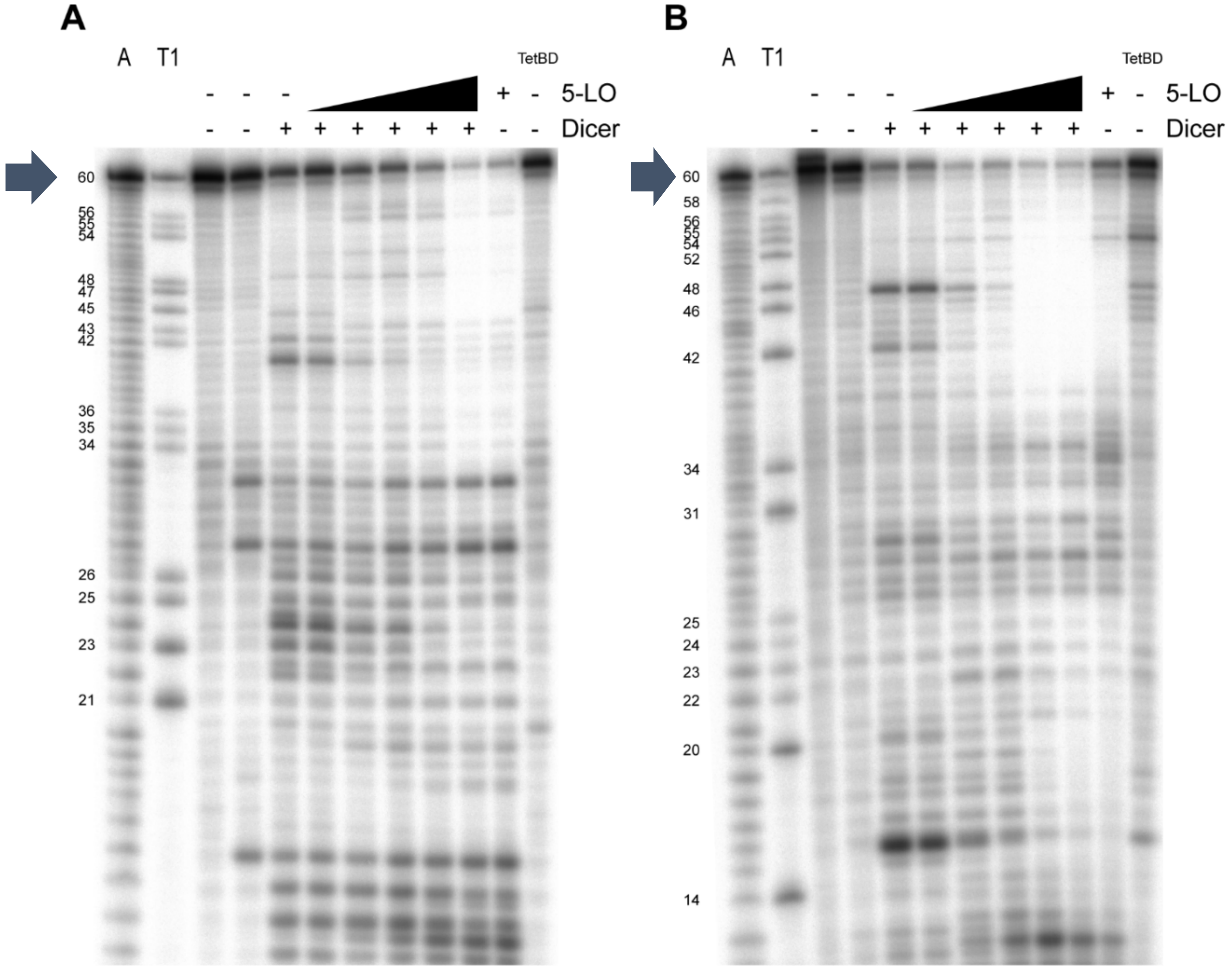

Fig. S5: 5-LO modulates pre-miRNA processing activity of human Dicer dose-dependently. Purified recombinant human Dicer 1650-1912 fragment (0.05 µM) was incubated at 21°C for 5 min without or with increasing amounts of recombinant human 5-LO (0.05, 0.625, 1.25, 2.5, and 5 µM) prior to addition of ^{32}P-labeled pre-miR-125a (**A**) or pre-miR-99b (**B**). After incubation at 37°C for 1 h samples were analyzed by denaturing PAGE and autoradiography. An alkaline (A, lane 1) and T1 ladder (T1, lane 2) were used as size markers. As control, pre-miRNA was loaded directly onto the gel without any incubation or after incubation with 5 µM 5-LO or 5 µM TetBD, respectively, as control. Full-length pre-miRs are marked by a blue arrow.

Table S1: Off-target analysis of the sgRNA used for the CRISPR-mediated knockout of 5-LO. Off-target analysis was performed by the CCTop - CRISPR/Cas9 target online predictor* (https://cctop.cos.uni-heidelberg.de:8043/index.html).

Coordinates	strand	MM	target_seq	PAM	distance		gene name	gene id
chr10:45382639-45382660	+	0	TGGATCA[CCGGCGATGTCG]	AGG	0	E	ALOX5	ENSG00000012779
chr16:3191351-3191372	+	4	TGCACCA[GCGGGGATGTCG]	GGG	14	I	AJ003147.9	ENSG00000262668
chr11:71554786-71554807	-	4	TAGAGCA[CAGGGGATGTCG]	TGG	5233	-	KRTAP5-9	ENSG00000254997
chr6:47402204-47402225	+	4	TGGGTCC[CCCGAGATGTCG]	GGG	32974	-	B3GNTL1P2	ENSG00000271328
chr18:79425653-79425674	+	4	TCGTTCA[CCGGCGGAGTCG]	GGG	2873	I	RP11-196B3.1	ENSG00000279637
chr19:10569232-10569253	+	4	TGGACCG[CCGGCAATGGCG]	GGG	173	-	CDKN2D	ENSG00000129355
chr13:66913491-66913512	+	4	TGTACCA[CCGGCTATGTAG]	GGG	886	I	PCDH9-AS2	ENSG00000228842
chr1:27593860-27593881	+	4	TGGGACA[CAGGCGATGTCT]	TGG	9516	I	AHDC1	ENSG00000126705
chr16:2578390-2578411	+	4	GGGACCA[CCGGCGGTGTGG]	GGG	0	E	RP11-20I23.10	ENSG00000280402
chr16:2621778-2621799	-	4	GGGACCA[CCGGCGGTGTGG]	GGG	0	E	CTD-3126B10.5	ENSG00000279057
chr9:96619211-96619232	+	4	CGGCTCA[CCGGTGATGTCC]	AGG	0	E	CDC14B	ENSG00000081377
chr8:1359984-1360005	-	4	TGAAACA[CCGGCGATGCTG]	AGG	8637	-	AF067845.1	ENSG00000260721

*Reference: Stemmer, M., Thumberger, T., del Sol Keyer, M., Wittbrodt, J. and Mateo, J.L. CCTop: an intuitive, flexible and reliable CRISPR/Cas9 target prediction tool. PLOS ONE (2015). doi: 10.1371/journal.pone.0124633
